# Differential Physiological Responses Elicited by Ancient and Heritage Wheat Cultivars Compared to Modern Ones

**DOI:** 10.3390/nu11122879

**Published:** 2019-11-26

**Authors:** Enzo Spisni, Veronica Imbesi, Elisabetta Giovanardi, Giovannamaria Petrocelli, Patrizia Alvisi, Maria Chiara Valerii

**Affiliations:** 1 Department of Biological, Geological and Environmental Sciences, University of Bologna, Via Selmi 3, 40126 Bologna, Italy; giovannam.petrocelli@studio.unibo.it; 2 Department of Medical and Surgical Sciences, University of Bologna, Via Massarenti 9, 40138 Bologna, Italy; veronica.imbesi@studio.unibo.it (V.I.); elisabett.giovanardi@studio.unibo.it (E.G.); chiaravalerii@hotmail.it (M.C.V.); 3 Pediatric Unit, Maggiore Hospital, Largo Bartolo Nigrisoli, 2, 40133 Bologna, Italy; patrizia.alvisi@ausl.bologna.it

**Keywords:** ancient and heritage wheat, gluten, immunogenic wheat peptides, gliadins, amylase trypsin inhibitors (ATI), celiac disease

## Abstract

Although ancient, heritage, and modern wheat varieties appear rather similar from a nutritional point of view, having a similar gluten content and a comparable toxicity linked to their undigested gluten peptide, whenever the role of ancient end heritage wheat grains has been investigated in animal studies or in clinical trials, more anti-inflammatory effects have been associated with the older wheat varieties. This review provides a critical overview of existing data on the differential physiological responses that could be elicited in the human body by ancient and heritage grains compared to modern ones. The methodology used was that of analyzing the results of relevant studies conducted from 2010 through PubMed search, by using as keywords “ancient or heritage wheat”, “immune wheat” (protein or peptides), and immune gluten (protein or peptides). Our conclusion is that, even if we do not know exactly which molecular mechanisms are involved, ancient and heritage wheat varieties have different anti-inflammatory and antioxidant proprieties with respect to modern cultivars. It is, therefore, reasonable to assume that the health proprieties attributed to older cultivars could be related to wheat components which have positive roles in the modulation of intestinal inflammation and/or permeability.

## 1. Introduction: Ancient, Heritage, and Modern Wheat Cultivars

Man first domesticated a diploid and tetraploid wheats about 10,000 years ago [1]. One of the wheat originally domesticated is sometimes called “einkorn,” and it has only one genome, designated as A genome. Since it is diploid, the plant genome designation is AA. The diploid wild wheat that was firstly domesticated was subjected to a continuous breeding and selection, which reasonably would have introduced and stabilized some new genes and/or controlling sequences into the genome. Consequently, the modern domesticated diploid wheat should have significantly different characteristics from the wild ones. The selection for the size of the seed, which occurred for a long time, certainly led to an increase in the starch content at the expense of the protein content [2]. The domesticated diploid wheat, einkorn, was designated *Triticum monococcum* by Linnaeus in the 18th century. The amount of *T. monococcum* that is grown today is very small and only partially used for human consumption. Tetraploid wheat was likely domesticated about the same time as diploid wheat [1]. It has two genomes designated as AABB. The A genome of the tetraploid wheats is closely similar to that of *T. monococcum*, while the B genome has been attributed to Triticum speltoides. The wild tetraploids are often designated Triticum dicoccoides, and the domesticated equivalents have been classified as *Triticum turgidum*. *T. turgidum* has two subspecies/varieties, one with the common name emmer and the other called durum. The main wheat of the Roman empire was emmer, and its modern cultivars are used for preparing human food, mainly biscuits and pasta. Durum wheats are now used mainly for pasta, although bread can be made from durum wheat, and such bread is still common in the south of Italy.

Hexaploid wheat has three genomes, designated A, B, and D. The A and B genomes of hexaploid wheats are nearly identical to the A and B genomes of tetraploid wheat. The hexaploid wheats have no wild equivalents and resulted from hybridization of a cultivated (domesticated) emmer and a wild grass species known as Triticum tauschii [3]. It was called *Triticum aestivum*, and its genome was designated AABBDD. These hexaploid wheats are free-threshing with the exception of spelt wheat, which has tightly adhering glumes and probably arose a few thousand years after the first wheat domestication.

There is evidence that the D genome of bread wheat has more epitopes active in Celiac Disease (CD) than the A and B genomes, and some of these showed, at least in vitro, to be the most active epitopes [4]. Consequently, diploid and tetraploid wheats (*T. monococcum*, for example) are likely to be less toxic to celiac patients than bread wheats. However, given that all of these wheats have proteins with several of the potentially active sequences that have been defined as toxic for people with CD [5,6], the significant decreased number of epitopes present in diploid and tetraploid wheats it is not sufficient to allow their consumption by affected subjects.

**Figure 1:**
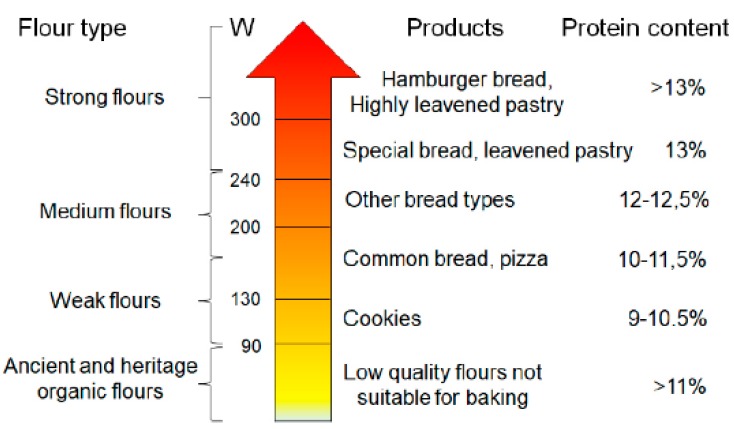
Flour quality, gluten strength (W), obtainable products, and protein content according to the parameters conventionally required by the food industry. Based on this classification, ancient and heritage wheat flours, obtained in organic farming, would not be suitable for baking since their W is commonly less than 90.

The protein and the gluten content of these wheats deserves a separate discussion. When grown today, the grains of the wild species usually have high protein contents, in the 16−28% range [2]. It is very likely that, following domestication, the protein content of wheats has steadily declined because of the increased starch content. Since the gluten content of wheat is approximately proportional to the protein content and usually ranges between 70 and 75% of total protein content, it seems that the earliest farmers were selecting seeds for lower protein and gluten content. Only with the development of yeast-fermented (leavened) bread baking about 2000−5000 years ago, farmers may have indirectly begun to select for higher protein content because leavened bread requires a relatively high gluten content: 10–11% of protein content is considered minimal for bread making. It is possible that this selection also increased the gluten strength of wheat, measured with the modern W index (Figure 1). Wheat cultivation methods, along with selection methods, remained substantially the same until the Second World War. The wheat cultivars introduced during the last centuries before the second World War are, therefore, defined as heritage varieties, thus diversifying them from the *ancient* cultivars that date back to the previous millennia. In addition, heritage varieties usually have higher protein content respect to modern ones [7].

After the second World War, two factors radically changed the methods of cultivation and selection of wheat. The first factor was the great availability of fertilizers (substantially nitrates and phosphates), deriving from the reconversion of the war industries, while the second historical factor is the beginning of the industrialization of the processes related to food production. From this moment the main criteria of grain selection become the high agricultural yield, obtained with the fertilization, and the high strength of the gluten, required to speed up the industrial procedures related to the processing of wheat. Another phenomenon that temporally occurred in the same period was wheat dwarfization [8], to avoid lodging and allow greater weight of the ear. The diminished height of the plant (from 150–180 to less than 50 cm) thus becomes the most evident characteristic that discriminates heritage wheat from those defined as modern varieties. The other major characteristic, less immediate to be measured, that discriminates between heritage and modern cultivars is the strength of the gluten, with the W index passing from values below 100 to values exceeding 300, which speed up both the formation of the dough and the pasta extrusion processes and at the same time it is responsible of the typical characteristics of the current wheat products, such as the elasticity of the breads (Figure 1) or the reduced gelatinization of starches during the pasta cooking [9].

## 2. Position of the Debate on Ancient, Heritage, and Modern Wheat Nutritional Proprieties

Wheat is the main staple food in many countries, providing a large percentage of daily energy intake for billions of people. Recently, ancient and heritage wheat cultivars have gained interest since several clinical studies have suggested that they could represent a healthier choice respect to modern ones (see Section 4). Although the majority of these studies do not concern patients affected by CD, it has been suggested that the increase in CD may be related in some way to a different gluten contained in modern grains [10]. That the serological prevalence of CD is increasing in the last 70 years, regardless of our greater capacity to diagnose it at least in industrialized countries, is a fact ascertained by independent studies carried out evaluating the CD positivity in blood banks [11,12,13]. Since the genetic predisposition cannot change in such a short period of time, the increased prevalence of CD in the last 50–70 years could be associated with the reduction of infant mortality rates due to the disease [14] or linked to changes in environmental exposures. Nutrition represents one of the major environmental factors, drastically changed during the last 70 years with the so-called westernization of the diet [15] but certainly not the only one. In addition to CD, non-celiac gluten sensitivity (NCGS) has recently appeared among the gluten-related diseases. NCGS was defined for the first time in a Consensus Conference in 2011 [16]. In addition, in this case, the spreading of the NCGS has been put in relation to the modern grains and to their different gluten [17], even if it is becoming clear that the gluten could not be the only responsible for the NCGS and, for this reason, some authors prefer to define it as non-celiac wheat sensitivity (NCWS) [18]. The current debate, therefore, concerns the possible differences between modern and heritage or ancient wheat cultivars with regard to human health and to wheat-related diseases, in particular. This has led to a dichotomy between scientists, separating those who believe that ancient grains have superior nutritional characteristics from those who believe that ancient, heritage, or modern wheat are all the same from a nutritional point of view. Thus, only starting from this two ideological points of view, we can explain the discrepancy between the conclusions of a study entirely carried out in vitro that states “beneficial properties claimed for old genotypes are not always sustainable with scientific evidence” [19] and the conclusions of another study, entirely conducted in vitro that finally “suggest the potential use of old wheat varieties…with health promoting characteristics” [20]. We are convinced that the nutritional characteristics of a cereal with a very complex composition, and that undergoes profound industrial transformations before being a food, are not easily deducible from single in vitro studies, and, for this reason, we propose an overall review of all the studies, including clinical trials on humans, to try to overcome an ideological separation that can be useful (and exploited) for the food industries but certainly not to the scientific community.

## 3. Differential Nutritional Aspects of Ancient and Modern Wheat Cultivars

### 3.1. Starch and Glycemic Index

The glycemic effects of whole and ancient cereals have been analyzed in several studies, both on animals and on humans. A generally accepted hypothesis is that the consumption of whole cereals can definitely improve their glycemic impact and thus reduce the risk of type 2 diabetes [21], even if some preclinical studies conducted on animal models of diabetes questions these benefits. For example, one of these studies compared the impact of 65% whole modern wheat consumption, with modern refined wheat consumption, considered as a negative control. The reported results showed that the consumption of whole modern wheat produced modest benefits in the development and progression of type 2 diabetes in these rats [22]. On the contrary, the introduction of whole wheat made from ancient cultivars (emmer and einkorn) seems to cause a downregulation of the key regulatory genes involved in glucose metabolisms and, as a consequence, a significant reduction in insulin levels in rats after nine weeks of intervention with emmer and einkorn [23]. Consistent with these preclinical results, clinical studies showed that the complete replacement of modern wheat with the heritage Khorasan wheat reduced fasting glucose and insulin levels both in healthy subjects and in persons with a high cardiovascular risk [24,25]. Other diets based on heritage varieties of soft hexaploid wheats, such as “Verna”, “Gentil Rosso”, and “Autonomia B”, resulted in a significant reduction in blood glucose after eight weeks of intervention in a double-blinded randomized clinical trial performed on 54 healthy subjects [26].

Since no differences have been found in the content of starch between heritage or ancient cultivars and modern ones, a hypothesis aimed at explaining the positive effects on the glycemic control of ancient and heritage cultivars is that they could have a different ratio between amylose and amylopectins within their starch [27], in analogy to what happens for the different rice cultivars [28]. This hypothesis, however, has never found sustenance in dedicated scientific studies. Thus, to date, we do not know exactly how ancient and heritage grains can improve glycemic responses, although the clinical studies clearly show this diversity. For example, it should be considered that the different industrial processes which ancient, heritage, or modern cultivar are generally subject to, can also have effects on postprandial glucose responses [29] (see Section 4).

### 3.2. Micronutrients

In scientific literature, there is a wide number of papers that shows how different is the phytochemical composition of the various cultivars of wheat (modern, heritage, and ancient). The phytochemicals and micronutrients content of wheat is strongly influenced by its genetics but also and profoundly by the environment and by the interaction between genetics and the environment. To compare different varieties of wheat, it is, therefore, necessary to cultivate them in the same environment and in the same year to minimize these important biases [30]. Agronomic and quality characteristics of old, heritage, and modern wheat varieties were analyzed in a study aimed primarily at organic farming [31]. The results show that the geographical area, the type of soil, and the climatic trend are the main variables that determine the different mineral content in the different wheat flours.

Recent studies carried out by Shewry and collaborators, within the “Healthgrain” project, compared cultivars of ancient and heritage wheat with modern ones, with regard to the content and composition of bioactive phytochemicals, such as phenolic acids, alkylresorcinols, tocols, sterols, folates, betaine, and choline. The results of these studies conclude that the ancient and heritage grains analyzed differ little from the modern cultivars in their content of most bioactive components and may even be lower in some components, such as dietary fiber [32,33,34].

Despite this, there are concordant data in the literature on the fact that ancient grains, such as Einkorn, Emmer, and Khorasan, have a higher content of carotenoids, particularly lutein, selected to give a yellow color to the grains and flour. Some einkorn cultivars showed lutein content values from three to eight times higher than modern soft wheat and twice higher than modern durum wheat [35,36]. The high content of lutein in einkorn could be significant for health in populations that consume it regularly, since it is characterized by anti-inflammatory and antioxidant properties, particularly exercised on the retina and on visual function [37]. Furthermore, ancient grains, on average, seem to contain greater amounts of selenium. This was highlighted in a study that focused on Khorasan wheat (Kamut^®^ brand) in which the highest selenium content was associated with its antioxidant and type 2 diabetes prevention properties demonstrated in this clinical study [38].

### 3.3. Polyphenols

Polyphenols are chemical compounds characterized by a phenolic ring and an organic carboxyl function. They are produced by plants (including wheat) as a physiological response against pathogens and represent the largest and most complex group of secondary metabolites present in cereals. The most common polyphenols in cereals are phenolic acids, which may be available in soluble forms (free compounds or conjugated with sugars) easily absorbed in the human intestine [35,39] or in insoluble forms, bound to the components of the cell wall. Other polyphenols include flavonoids (proanthocyanidins and their glycosylated form, anthocyanin), alkylresorcinols, and lignans [40]. Several studies have shown a protective role of polyphenols in human health and, in particular, against cardiovascular diseases, diabetes, and the metabolic syndrome [41]. A study performed in 2015 [33] summarizes several data obtained from the analysis of ancient and modern varieties of different compounds, including phenolic acids. The comparison between modern (soft) wheat and ancient cultivars (einkorn emmer and spelt) showed a higher average concentration of phenolic acids in ancient varieties. However, the authors conclude that due to the high variability between samples and in the analysis methods, these differences should not be considered truly significant. A study conducted in 2011 analyzed the polyphenol content of 16 heritage cultivars and six modern wheat varieties [42], cultivated in the same geographical location, showing a different polyphenol content profile, with a greater number of total compounds and isomers observed in six heritage varieties compared to modern ones. In particular, the Verna, Canove, Carosello, Gentil Rosso, Gentil Rosso Mutico, and Sieve cultivars showed a higher free polyphenol content than the average measured values, while the higher polyphenol content was found in the Carosello, Marzuolo D’Aqui, Marzuolo Val Pusteria, Gentil Rosso, Inallettabile, and Verna varieties but also in two modern varieties, Eureka and Nobel. The highest value of free flavonoids was found in five heritage varieties (Andriolo, Gentil Rosso Mutico, Marzuolo D’Aqui, Sieve, and Verna), while the highest values for bound flavonoids were found in the Gentil Rosso, Gentil Rosso Mutico, Marzuolo D’Aqui, Marzuolo Val Pusteria, and Inallettabile and in two modern varieties, Eureka and Nobel [42]. Another study in 2019 [43] evaluated the content of free soluble phenolic compounds of eight heritage varieties (Autonomia, Gentil Rosso, Inallettabile, Leone aristato, Mentana, Poulard di Ciano, Risciola, and Terminillo) and two modern ones (Bolero and Blasco). The results showed a higher concentration of phenolic compounds in the heritage varieties Terminillo, Risciola, Gentil rosso, Mentana, and Leone aristato [43]. Although further analysis are needed on a larger number of samples and with standardized methodologies, the overall data suggests that ancient and heritage are qualitatively superior to modern varieties in this aspect. Nevertheless, considering the role that wheat consumption plays in the human diet, and, in particular, the many other sources of polyphenols present in the Mediterranean diet (in fruits and vegetables), the differences observed between wheat varieties may not be relevant in the context of the overall human diet.

### 3.4. Lipid Profiles

It has been hypothesized that one of the reasons for the improvement of the lipid profile induced by a diet based on ancient or heritage cultivars in patients suffering from different pathologies is the better lipid profile of these cultivars, and, in particular, their higher content in mono- and polyunsaturated fatty acids (MUFA and PUFA) [27]. Although the lipidomics of the grains is rather complex and not easily simplified with the MUFA and PUFA content, there are experimental evidence that ancient wheats, such as einkorn, emmer, and spelt, have characteristic lipidomic profiles that are different from each other [44]. A study carried out using modern and heritage wheat varieties has revealed differences between the lipidomic profile of soft grains (Blasco, Bologna, and Virgilio) and that of durum cultivars (Odisseo, Timilia, Senatore Cappelli, and Miracolo) [45]. However, this study did not reveal significant differences in the lipid profile between heritage (Virgilio, Timilia, Senatore Cappelli, and Miracolo) and modern grains (Blasco, Bologna, and Odisseo), while it did not analyze any ancient varieties [45]. It must be considered that the geographical and agronomic conditions can probably influence the lipid profile of the wheat. For example, it has been ascertained that nitrogen fertilization is able to modify the total lipid content and the lipid composition of wheat [46]. Despite this, we can conclude that to date there is no scientific evidence of differences in lipid composition between heritage and modern cultivars, while extensive comparative studies of the differences in the lipid profile between ancient and modern wheats are still lacking. Overall, we can conclude that lipids represent a minor nutritional component of wheat, despite being important contributors to flour processing and, particularly, for bread making. In human nutrition, the lipid contribution of cereals is certainly a minority one and it is, therefore, unlikely that differences on this specific aspect can modify the overall intake in MUFA and PUFA of a sufficiently varied human diet.

## 4. Different Food Processing for Different Wheats

With the industrial revolution, wheat-based natural food started to be more and more processed [47]; today, transformations are become so drastic that highly processed wheat-based products are routinely consumed in our diet. Since our genetic background is not really adapted to these severe diet transformations, our physiology is not completely adequate to tolerate the food we eat. For these reasons, wheat-related disorders have been attributed to these changes: our organism and digestive physiology could be not adapted to wheat-based foods [48]. In addition to refining, among drastic and less natural processes applied to wheat, we can also cite higher kneading intensities for bread baking, the use of baking powder instead of natural leaven, the increased use of extrusion-cooking at high temperature, and an increased use of additives, such as the vital gluten. Starting from the beginning, the industrial processes that applied to wheat was the milling. Most of the modern wheat undergoes a cylinder milling, which leads to the production of refined flours of type 0 and 00 almost totally deprived of the fiber and the vitamins contained in wheat germ. On the contrary, a good part of the ancient and heritage wheats are milled in millstones and give rise to wholemeal or less refined type 1 and 2 flours. In industrial processes for bread baking, leavening powders are often used instead of natural yeasts. Natural leavening increase digestibility of wheat proteins, including gluten [49] and other proteins with potential inflammatory effects, such as amylase trypsin inhibitors (ATI) [50], potentially reducing their immunogenic load (see Section 5 and Section 6). In bakery products, vital gluten (i.e., ‘exogenous’ gluten) can be added to confer technological properties, such as emulsification, cohesiveness, viscoelasticity, gelation, and foaming. It has been calculated that the intake of vital gluten has tripled between 1977 and 2012, from 140 to more than 400 g/person/year in the USA [10]. Finally, the higher temperature industrially adopted for fastening bread baking or pasta drying generally decreases the wheat protein digestibility [49]. What we can say to highlight differences between products based on modern wheats and those based on ancient or heritage ones is that modern wheat are much more frequently subjected to strong industrial processes, while ancient and heritage cultivars are often processed by using more traditional methods. This is obviously not a rule, but it is what frequently happens. The ancient or heritage cultivars are often stone-milled and, for the preparation of the bread, they are leavened using traditional yeasts (*S. cerevisiae*) or even the sourdough, very rich in lactobacilli capable of effectively degrading one of the inflammatory components of the wheat proteome: the ATI proteins [50]. Interestingly, sourdough baking seems to reduce the quantities of both ATIs and fermentable oligo-, di-, mono-saccharides and polyols (FODMAPs), short chain carbohydrates present in wheat that are poorly absorbed and contribute to intestinal bloating [51]. On the other hand, modern grains are often refined and used for the production of processed or ultra-processed foods, with the addition of additives, such as vital gluten, which drastically worsen their nutritional values, sometimes making them fall into the worst category of the NOVA classification for processed food (NOVA 4) [52]. These industrial processes obviously do not depend on the intrinsic nutritional values of the different wheat cultivars but can certainly improve or worsen their nutritional properties.

## 5. Studies on Gluten Immune Toxicity

Gluten (from Latin gluten, “glue”) is made by a group of proteins, called gliadins and glutenins, which localize with starch in the endosperm of wheat. Gluten forms only when glutenin molecules cross-link via disulfide bonds to form a submicroscopic network attached to gliadin, into the dough [53]. In 2010, van den Broeck and collaborators first analyzed the presence of two gliadin epitopes toxic for CD patients, defined as Glia-alpha9 and Glia-alpha 20, in a wide selection of ancient, heritage, and modern wheat cultivars [54]. The modern grains analyzed were originating after the 1980s. The results of their study were that the Glia-alpha9 epitope was actually more represented in modern cultivars, despite a rather variable presence within the single groups: ancient, heritage, and modern ones. The study is certainly remarkable for the large number of cultivars analyzed, while it is certainly reductive due to the low number of gluten “toxic” epitopes that were analyzed. A more recent study, conducted by Ribeiro and coworkers in 2016 [55], broadens the spectrum of toxic peptides analyzed to five. The study analyzes a total of 126 cultivars, divided between ancient, heritage, and modern. The conclusions of this study were that, on average, the number of toxic epitopes analyzed decreased in the modern soft wheats compared to the ancient soft ones, while substantially it remained unaffected within the group of durum wheats. The researchers’ conclusions were, therefore, that the breeding that the wheat has undergone in the last 100 years did not seem to have increased the quantity of gliadin toxic epitopes. This conclusion would be also supported by the fact that the selection for a high gluten strength (W) seems to have acted more on glutenin genes, while most of the peptides toxic for CD patients have been found in the gliadins. The study conducted by De Santis and collaborators [56] focuses on the comparison between heritage cultivars released from 1900 to 1949 and modern cultivars obtained from 1985 to 2005 with regard to both glutenin and gliadin content, and with regard to the presence of alpha and gamma gliadins, considered the most toxic proteins for CD patients. The results of this study were that in modern cultivars the total amounts of alpha and gamma gliadins did not change, even if a decrease in omega-5 gliadin was observed. In these modern wheats, however, the glutenin content increased, with the gliadin/glutenin ratio decreasing from 2.8 to 1.7. One of the hypotheses that has been proposed is that the selection for gluten strength may have reduced its digestibility. In fact, the gluten peptides considered “toxic” are formed exclusively during gastric end enteric digestions: If gliadins and glutenins were degraded to di- or tri-peptides, their immunogenic determinants would disappear. This hypothesis was firstly tested in a proteomic study [57] that compared five modern cultivars with five heritage cultivars and only one ancient variety: einkorn (*T. monococcum*). The result of this study is that the number of gluten “toxic” peptides, after in vitro digestion, is considerably lower in einkorn, while the heritage varieties analyzed produced a higher quantity of peptides containing immunogenic and toxic sequences than modern ones. The results for diploid wheats appear to be different, and the reduced immuno-toxicity of *T. monococcum* has been demonstrated in another study that confirms the better digestibility of this cereal in vitro gluten [58]. Recently [19], a new study, based on in vitro digestion, has been carried out on nine heritage cultivars, released between the early 1900s and 1950s, compared with three modern cultivars obtained after 2004. This study concluded that the digestion of old genotypes generally yielded peptides in greater concentration. In particular, five peptides of γ-gliadin, known to trigger the adaptive immune reaction, and two peptides of α-gliadin, known to be toxic to celiac patients, were particularly abundant in some heritage varieties.

It should be emphasized that in vitro digestion systems are models that do not succeed in reproducing exactly what happens in the intestine. Moreover, since wholemeal flour was subjected to simulated gastrointestinal digestion, the gluten network was never really studied in these in vitro digestion experiments. Nevertheless, the hypothesis of a better digestibility of gluten does not seem to be supported, at least for the heritage varieties.

In conclusion, the studies carried out on the “toxicity” of gluten, before and after in vitro digestion, indicate a superiority of ancient diploid varieties (*T. monococcum*, in particular) but an equal or greater toxicity of heritage cultivars with respect to the modern ones. It should be added that these studies concern only one of the gluten-related diseases, namely CD, and that the internationally accepted guidelines currently provide that these patients should, however, avoid any type of wheat or cereal containing gluten, including einkorn.

## 6. Whole Wheat Proteome Immunogenicity

As well as CD is not the only gluten-related disease, gluten are not the only group of wheat proteins to have immunogenic and potentially negative effects on the intestine. Wheat amylase and trypsin inhibitors (ATIs) represent a family of up to 17 proteins with molecular weights between 12 and 15 kDa and a variable primary but conserved secondary structure characterized by five intrachain disulfide bonds and alpha helices. ATIs represent up to 4% of total wheat protein in *T. aestivum* (hexaploid) and are highly resistant to intestinal proteases [59]. They are capable of activating Toll Like Receptor 4, (TLR4), a pro-inflammatory receptor activated by Lipopolysaccharide (LPS) and other toxins [60]. Relevant biological activity is confined to ATIs in gluten-containing cereals, while gluten-free cereals display no or minimal activities. ATIs may also act as interfering contaminant of gluten and have been identified to play a role in the occurrence of a broad range of disorders, including CD, non-celiac gluten sensitivity (NCGS), and functional gastrointestinal disorders [51,61,62].

In modern hexaploid wheat species, up to 19 different encoding genes for ATIs and similar protease inhibiting proteins have been identified by complete genome sequencing [63], of which 13 have been evidenced at protein level in wheat. Two of the most active ATIs, called 0.19 and CM3, showed to have the highest bioactivity on TLR4-bearing monocytes when extracted from modern exaploid cultivars, whereas ATI extracts from the hulled wheat species spelt (*T. spelta*, hexaploid), emmer (*T. dicoccum*, tetraploid) showed to have a reduced activity [64]. Furthermore, liquid chromatography-tandem mass spectrometry (LC−MS/MS) confirmed that einkorn (*T. monococcum*) contained very low amounts of ATIs or even none. Even if data on expression and activity of ATIs in heritage wheat are lacking, what we know so far is that the expression of ATIs (and their variety) seems to increase with ploidy and is, therefore, greater in hexaploids (*T. aestivum*) than in tetraploids (*T. durum*), while diploids (einkorn) have a very low ATIs expression. In an in vitro study on ATIs activity, measured as IL-8 secretion in myeloid cells, it was shown that spelt (*T. spelta*, hexaploid) could be an exception since authors detected lower, even if not significant, ATIs activity in this ancient hulled wheat cultivar [62]. A recent proteomic study on ATIs expression casts doubt on this datum and instead measured a higher expression of ATIs in spelt than in other modern hexaploid cultivars [65]. However, this work also confirms the evolutionary increase of the total expression of ATI proteins with increasing ploidy.

The inflammatory potential of the total wheat proteome was tested in vitro, using peripheral blood leukocytes obtained from patients diagnosed with NCGS. In a first study [17], two heritage varieties were compared with two modern ones, and a lower synthesis of a chemokine related to the intestinal permeability and inflammation (CXCL10) was observed in leukocytes treated with heritage cultivar extracted proteins. One of this cultivar was the Khorasan wheat (*T. turanicum*, tetraploids), where ATIs proteins resulted to have a lower pro-inflammatory activity in myeloid cells in the study by Zevallos and coworkers [62]. Similar results on CXCL10 secretion were obtained in a second study on pediatric NCGS patients [66], in which two heritage varieties were compared to a modern one.

## 7. Wheat Cultivars and the Microbiome

Intestinal human microbiota is involved in several metabolic processes besides the well-known role in gut-associated immune system homeostasis. Wheat proteins, such as gluten and ATI, are only partially digested by intestinal proteases but can be effectively degraded by intestinal microbiota in a complex interaction that can have an impact on both peptide immunogenicity and microbiota composition and that certainly affect the risk of developing disorders related to wheat (A). Indeed, it has been demonstrated that wheat peptides after microbial digestion can have an increased or reduced immunogenicity depending on the degrading bacteria. *Pseudomonas aeruginosa*, an opportunistic pathogen detected in CD patients, is able to further degrade protease-generated gluten peptides in smaller fragments with immunogenic sequences and a higher ability to translocate trough the epithelial barrier and thus interact with the gut immune system. On the other hand, *Lactobacillus* spp., which is highly represented in healthy subjects, is able to decrease the immunogenicity of wheat peptides generated by human or bacterial digestions [67,68]. Partially digested gluten peptides constitutes a substrate for other gut bacteria, such as *Rothia *spp. [69]. Since it is well known that the intestinal microbiota is susceptible to food components, the general quality of food and the specific quality of wheat could affect the gut microbiota ecology. Human studies showed changes in the amount of bacterial family and species linked to whole wheat consumption. In particular, an increase in *Bifidobacterium*, *Lactobacillus*, *Enterococcus*, and *Prevotella* species [70,71] and a decrease of *Dialister*, *Blautia*, and *Collinsella* [71] have been linked to whole wheat consumption, even if no changes resulted at genus or higher taxonomic levels [71,72]. However, while providing interesting data taken as a whole, these studies show a strong heterogeneity in the experimental design and in the methods used, as pointed out by Koecher and collaborators [73]. As for heritage grains, a single study showed an overall positive effect of Khorasan whole wheat on the microbiota, when compared to modern whole wheat. In particular, in healthy volunteers, the Khorasan-based diet showed a tendency to decrease in *Bacteroides*/*Prevotella*, associated with an increased release of short chain fatty acids [74]. The role of the consumption of whole wheat and, in particular, of ancient or heritage cultivars on the microbiota ecology must be further studied in vivo, above all focusing on the pathological conditions, and, in particular, the functional intestinal disorders and the wheat-related diseases.

An in vitro, study conducted on soluble fiber extracted from heritage or modern wheat varieties has attempted to highlight differences in their prebiotic properties. Soluble fibers were added to cultures of *B. pseudocatenulatum* and *L. plantarum*, and the results showed that both the soluble fiber of heritage and modern cultivars had a good prebiotic activity [75]. An in vivo study conducted on pigs, fed for 30 days with a diet including *T. monoccum* or standard modern wheat, showed changes in the microbiota of pigs fed with this ancient cultivar. In particular, an enrichment in the short-chain fatty acids producing bacteria (genera *Blautia, Faecalibacterium*, and *Oscillospira*) associated with an increased metabolic and microbial diversity was observed in pigs fed with *T. monococcum* but not in those fed with modern wheat, suggesting a strong beneficial prebiotic effect of this ancient variety on the health of the intestinal ecosystem [76].

## 8. Ancient and Heritage Wheat in Clinical Study

To date, there are some relevant studies conducted on human beings aimed at testing the effect of consumption of different varieties of wheat, and, in particular, heritage and ancient, on diabetes, cardiovascular diseases, Nonalcoholic Fatty Liver Disease (NAFLD), and gastroenteric disorders, such as Irritable Bowel Syndrome, Non-Celiac Gluten Sensitivity, and also CD. In all these studies, enrolled patients were asked to replace all the grain sources usually consumed with those specifically intended for the study, which were well characterized and standardized in terms of quantity and nutritional properties. Many of these studies have been focused on the heritage variety Khorasan (Kamut^®^ brand). This is due to the fact that this multinational company has invested heavily in terms of scientific research. However, this does not diminish the importance of these studies or their scientific value. A pilot study conducted on 21 patients with type 2 diabetes mellitus (T2DM) showed that the replacement of modern wheat with Khorasan wheat for eight weeks was able to significantly reduce insulin and glucose levels with respect to modern wheat consumption [77]. In 2017, a second study carried out on 30 healthy volunteers confirmed the nutritional properties of Khorasan. Healthy volunteers were randomized to follow a Khorasan-based diet or a modern grain-based diet [38]. After 16 weeks, the group following the Khorasan-based diet had significantly reduced fat mass and blood insulin levels, while showing higher levels of docosahexaenoic acid (DHA), a polyunsaturated fatty acid considered protective against the onset of diseases, such as diabetes, coronary heart disease, and even cancer [78,79,80]. A randomized crossover study of 22 healthy subjects demonstrated a protective role for Khorasan wheat consumption in cardiovascular disease. Eight weeks of diet based on this heritage cultivar resulted in a significant reduction in total cholesterol, LDL, and glucose [24] in the blood. A decrease in triglycerides and fasting plasma glucose was observed in a randomized, crossover clinical trial on 63 healthy volunteers [81]. Furthermore, a randomized crossover study conducted on 22 patients suffering from acute coronary syndrome confirmed the results, showing again a decrease in blood levels of total cholesterol, LDL-C, fasting glucose, and insulin that were not observed after the consumption of modern cultivars [25].

A particularly interesting study involved 40 patients diagnosed with NAFLD [82]. In this randomized double-blind study, patients followed a diet based on Khorasan wheat as the sole source of wheat (pasta, bread, crackers, biscuits) or a diet based on modern grains (pasta, bread, crackers, biscuits) for a period of three months. The results of this study showed that in the Khorasan group alone there was a significant decrease in alanine aminotransferase (ALT), aspartate aminotransferase (AST), and alkaline phosphatase blood levels, three significant markers of liver function. Furthermore, cholesterloemia was also found decreased in the Khorasan group. From an inflammatory point of view, however, significant decreases were observed in some pro-inflammatory circulating cytokines, such as IL-1ra, IL-8, and Tumor necrosis factor alpha (TNF-α), in NAFLD patients after the dietary intervention with the Khorasan heritage wheat [82]. A systemic anti-inflammatory effect of Khorasan wheat consumption was also detected by our group in a recent randomized crossover pilot study involving 20 professional or semi-professional athletes [7]. In our study, after four weeks of a Khorasan-based diet, a significant reduction in blood levels of the Monocyte chemoattractant protein 1 (MCP-1), a pro-inflammatory chemokine produced by muscle during strenuous activity, was recorded.

One of the most interesting studies has compared the effects of nutrition based on three heritage cultivars (Verna, Gentil Rosso, and Autonomia B) to that based on a modern wheat (Blasco) [26]. In this study, 45 healthy subjects were randomized into groups fed with wheat from a single cultivar. Furthermore, this study compared the same cultivars produced by conventional agriculture and by organic farming. The results of this study demonstrate once again that the consumption of heritage varieties has led to a significant blood reduction of total cholesterol, LDL cholesterol, and glucose, which was not registered after the modern wheat-based diet. It is interesting to note that no experimental differences were found between the grains from organic cultivation and those obtained with conventional agriculture and that, therefore, the differences were attributable to different genetic and nutritional characteristics of the grains. Two human studies instead focused on gastrointestinal disorders, particularly on irritable bowel syndrome (IBS) and CD. The first study was a randomized double-blind study of 20 subjects who were randomly assigned to switch from the modern wheat-based diet to a Khorasan-based diet. During the intervention period with Khorasan wheat-based products, IBS patients showed a significant reduction in symptom severity associated with a decrease in serum levels of pro-inflammatory cytokines, such as IL-6, IL-17, interferon-γ, and MCP-1, cytokines and chemokines that in some studies were found to be higher in the IBS patient population than in healthy controls [83]. The second clinical study instead involved only eight CD patients which were following a gluten-free diet and were asked to eat biscuits made with the ancient wheat *T. monococcum* for 60 days [84]. Although the study confirmed the toxicity of this variety for patients with CD from a histological and serological point of view, this ancient variety of wheat was clinically well-tolerated, suggesting a potential efficacy in patients suffering from other gluten-related diseases, such as NCGS.

Table 1 summarizes all the clinical trial so far conducted on ancient or heritage wheat cultivars. At clinical level, these studies, in agreement with each other, demonstrate that the main effects on humans of which these grains are capable seem to be those antioxidants and anti-inflammatories, which as a consequence lead to beneficial effects on metabolic and clinical parameters.

## 9. Conclusions and Future Directions

The analysis of recent literature makes clear differences between the so-called ancient grains, those released before the 1950s (defined as heritage), and the modern cultivars. From the macro- and micronutrient composition point of view, the studies conducted so far do not reveal huge differences between the wheat cultivars of different ages, even if the content in secondary metabolites and in polyphenols seems higher and more varied in older cultivars. In addition, with regard to micronutrients, various studies suggest that there may be differences, especially in terms of mineral content, with an advantage of older wheats. As far as the protein component is concerned, ancient or heritage cultivars tend to have, at the same crop conditions, a higher content in proteins, and, therefore, in gluten, but they have a completely different gluten quality with a very weaker structure and strength. In fact, the W values of the modern wheats are always 2–3 times higher than all their predecessors, since the high W value has been one of the central criteria that guided the wheat selection from 1940–50 onwards. Despite this big evolutive diversity, there does not seem to be any difference between heritage and modern wheats in the production of immunogenic and toxic gluten peptides, although these type of in vitro studies have non-negligible biases. The ancient grains instead, and, in particular, the *T. monococcum*, showed a less toxic and more digestible gluten, although they remain still ineligible for CD patients. In addition, with regard to the ATI immunogenic proteins and peptides, there is no doubt that these are much less expressed in ancient grains, while it is still controversial to consider the fact that they could also be less expressed in heritage cultivars.

Despite these differences, which appear quite modest at the nutritional level, when the flours or their extracts are added to cell cultures, the differences between ancient or heritage grains and modern ones become more easily detectable [85]. When switching to clinical trials, performed on patients and on healthy subjects, all doubts disappear and diets based on ancient or heritage cultivars always showed clear advantages in terms of anti-inflammatory and antioxidant activities. The limit of these studies are certainly the low number of cultivars tested so far and, sometimes, the low number of subjects enrolled. Despite this, considering the results of all the available trials (Table 1), it could be said that modern grains, when clinically tested, clearly showed pro-inflammatory and pro-oxidant activities even if, from a molecular point of view, we do not yet know what these negative health activities are due to. Continuing to affirm that, from a nutritional point of view, modern, ancient, or heritage wheats are all the same, means deliberately ignoring all the clinical studies conducted so far.

From a commercial point of view, these healthier nutritional properties of ancient and heritage grains have been overly exploited, even though in many countries there are no wheat chain controls aimed at guaranteeing consumers that the grains indicated as heritage or ancient on the packaging of the products are really of those types. Furthermore, the quantities of ancient and heritage wheats really produced would probably not guarantee the current production of self-declared “ancient wheat based” foodstuff.

## Figures and Tables

**Table 1:** Human intervention trials on ancient or heritage cultivars performed on healthy or diseased subjects.

Inclusion Criteria	Experimental Design	No. of Subject Enrolled	Ancient/Heritage Wheat Varieties	Control Wheat	Duration of Intervention	Recorded Effects (Ancient/Heritage vs. Control)	Ref.
Type 2 Diabetes Mellitus.	Randomized, parallel groups. Enrolled patients were randomized to follow an ancient/heritage grain-based diet or a modern grain-based diet.	**22** (8 m, 14 f), age: 42–71 years.	Whole grain flour: *Triticum dicoccum.*	Whole grain flour: *Triticum aestivum (modern)*.	6 weeks.	Decreased blood total cholesterol, triglycerides, and LDL-cholesterol.	[86]
Healthy subjects.	Open label.	**11** Men, mean age: 25 ± 2.	*Triticum monococcum* (Einkorn Bread).	*Triticum aestivum (modern)*.	One single meal test.	Decreased Gastric Inhibitory Peptide (GIP) with einkorn bread.	[87]
Healthy subjects.	Non-blinded, crossover. Enrolled patients were randomized to follow a heritage grain-based diet or a modern grain-based diet.	**20** (11 m, 9 f), median age 39.5 years.	Semi-Whole grain: *Triticum aestivum* Verna.	Semi-Whole grain: *Triticum aestivum* mixed modern varieties.	10 weeks of diet intervention.	Decreased blood total cholesterol, LDL-cholesterol, IL-8, whole blood viscosity.	[88]
Baker’s asthma or wheat allergy.	Interventional open label: Oral and bronchial challenges, prick test.	**66** (45 m, 21 f), mean age: 28.6 ± 12.9 years.	*Triticum spelta* grain extracts.	*Triticum aestivum* grain extract.	Immediate.	Decrease of wheal area and percentage of positive challenge tests.	[89]
Healthy subjects.	Randomized, single blind, crossover. Enrolled patients switched from heritage grain-based diet to modern grain-based diet.	**22** (8 m, 14 f) mean age: 50.5 ± 11.8 Years.	Semi-Whole grain: *Triticum turgidum*. Khorasan.	Mix of semi-Whole grain: *Triticum durum* and *Triticum Aestivum* (modern).	8 weeks diet intervention.	Decrease of blood total cholesterol, LDL-cholesterol, glucose, TNFα, IL-6, IL-12, and Vascular endothelial growth factor (VEGF). Increase: K^+^, Mg^2+^.	[24]
Healthy subjects.	Randomized, single-blinded, crossover trial. Enrolled patients switched from heritage grain-based diet to modern grain-based diet.	**20** (11 m, 9 f), age: 21–61 years. Mean BMI: 26.1 ± 2.5 (m) 24.8 ± 4.9 (f)	Semi-whole grain: *Triticum durum* Senatore Cappelli.	Mixed modern semi-whole grain *Triticum durum* wheat varieties	10 weeks diet intervention.	Decrease of total cholesterol, whole blood viscosity, and Red Blood Cell deformability.	[90]
Celiac Disease (CD) patients.	Single blind, crossover trial.	**12** (4 m, 8 f) mean age: 44.5 ± 10 years.	*Triticum monococcum*.	Amygluten (pure gluten) and rice.	Administration of a single dose.	No significant conclusions.	[91]
Irritable Bowel Syndrome (IBS) patients (Rome III Diagnostic Criteria).	Randomized, double-blinded, crossover trial. Enrolled patients switched from ancient/heritage grain-based diet to modern grain-based diet.	**20** (7 m, 13 f), median age 35.5 years.	Semi whole grain: *Triticum turgidum* Khorasan.	Modern Italian durum and soft wheat varieties.	6 weeks diet intervention.	Decrease in the severity of IBS symptoms (abdominal pain, bloating, stool consistency, and tiredness). Decrease of IL-6, IL-17, Interferon gamma (IFN-γ), Monocyte chemoattractant protein 1 (MCP-1), VEGF.	[83]
Healthy subjects.	Parallel arms.	**30** (4 m, 26 f), mean age: 37 ± 7 years.	Whole grain *Triticum turgidum* Khorasan.	whole grain: *Triticum durum*.	3 months.	Increase of short chain fatty acids (SCFA), phenol compounds and an increase in health-promoting mutualists in the gut microbiota.	[74]
Type 2 Diabetes Mellitus.	Randomized, double blind, crossover. Enrolled patients switched from ancient/heritage grain-based diet to modern grain-based diet.	**24** (14 m, 7 f), mean age 64.4 ± 10.9 years.	Semi-whole grain: *Triticum turgidum* Khorasan.	Mix of semi-whole grain: *T. durum* and *T*. *aestivum* (modern varieties).	8 weeks diet intervention.	Decrease of blood total and LDL cholesterol, insulin and fasting glucose. Decreased levels of reactive oxygen species (ROS), VEGF, and IL-1ra.	[77]
Acute Coronary Syndrome.	Randomized, double-blinded, crossover trial. Enrolled patients switched from heritage grain-based diet to modern grain-based diet.	**22** (13 m, 9 f), median age 61 years.	Semi whole grain: *Triticum turgidum* Khorasan.	Mix of semi-whole grain: *T. durum* and *aestivum* (modern).	8 weeks diet intervention.	Decrease of blood total cholesterol, LDL-cholesterol, fasting glucose, insulin ROS, and TNF-α.	[25]
Healthy young athletes.	Randomized, single-blind crossover trial. Enrolled patients switched from ancient/heritage grain-based diet to modern grain-based diet.	**20**, men, median age 18.3 (15–25) years.	Semi whole grain: *Triticum turgidum* Khorasan.	Semi-whole grain: *Triticum turgidum* ssp. *durum*, *Triticum aestivum* (modern).	4 weeks diet intervention.	Decrease of MCP–1 and improvement of self–rated health status.	[7]
CD patients in remission and in GFD diet.	Phase II, open label.	**7** 1 man, 6 women, median age 37 ± 7.3 years	*Triticum monococcum*.	None.	60 days.	Increased villous atrophy and recurrence of dermatitis herpetiformis.	[84]
Healthy subjects.	Randomized, double-blinded, crossover trial. Enrolled patients switched from heritage grain-based diet to modern grain-based diet. Three diet intervention studied: 1. Verna 2. Blasco 3. Gentil Rosso or Autonomia B.	**45** 32 men, 13 women, median age 50.1 (25–75) years.	*Triticum aestivum*: Verna, Gentil Rosso, Autonomia B.	*Triticum aestivum*: Blasco (modern).	8 weeks diet intervention.	Decrease of blood total cholesterol, LDL-cholesterol, and fasting glucose.	[26]
Pre-hypertensive non diabetic adult volunteers with no metabolic, cardiovascular, gastrointestinal, nor endocrine major disorders.	Double-blind, randomized, feeding-controlled, crossover. Enrolled patients switched from heritage grain-based diet to modern grain-based diet.	**63** (30 m, 33 f), mean age 55.9 ± 6.8 years.	*Triticum turgidum* Khorasan.	Mix of *T. durum* varieties and *T. aestivum* (modern).	4 weeks diet intervention.	Decrease of blood triglycerides, fasting glucose, systolic blood pressure, and rise of pulse volume change.	[81]
Nonalcoholic Fatty Liver Disease (NAFLD)—mild to moderate liver steatosis—NO, excessive alcohol consumption, type 2 diabetes mellitus (T2DM), viral hepatitis, NASH, and chronic liver diseases.	Randomized, double-blinded trial with two parallel arms. Enrolled patients were randomized 1:1 to follow an heritage grain-based diet or a modern grain-based diet.	**40** (12 m, 28 f), mean age 55.2 ± 10.4 years.	Semi whole grain: *Triticum turgidum* Khorasan.	Mix of semi-whole grain: *T. durum* and *T.* aestivum (modern).	3 months.	Decrease of alanine aminotransferase (ALT), aspartate aminotransferase (AST), TNF-a, IL-1ra, IL-8, and IFN-γ.	[82]
Healthy subjects.	Randomized, non-blind, parallel arm study. Enrolled patients were randomized 1:1 to follow an ancient/heritage grain-based diet or a modern grain-based diet.	**30** median age 37 years.	Whole grain: *Triticum turgidum* Khorasan.	Mix of whole grain modern commercial Italian durum wheat.	16 weeks.	Decrease of fat mass and blood insulin; increase of docosahexaenoic acid (DHA).	[38]
